# Alternative initiation and splicing in dicer gene expression in human breast cells

**DOI:** 10.1186/bcr1043

**Published:** 2005-05-16

**Authors:** Charletha V Irvin-Wilson, Gautam Chaudhuri

**Affiliations:** 1 Division of Cancer Biology, Department of Biomedical Sciences, Meharry Medical College, Nashville, TN 37208, USA

## Abstract

**Introduction:**

Dicer is a ribonuclease that mediates RNA interference both at the transcriptional and the post-transcriptional levels. Human dicer gene expression is regulated in different tissues. Dicer is responsible for the synthesis of microRNAs and short temporal (st)RNAs that regulate the expression of many genes. Thus, understanding the control of the expression of the dicer gene is essential for the appreciation of double-stranded (ds)RNA-mediated pathways of gene expression. Human dicer mRNA has many upstream open reading frames (uORFs) at the 5'-leader sequences (the nucleotide sequence between the 5'-end and the start codon of the major ORF), and we studied whether these elements at the 5'-leader sequences regulate the expression of the dicer gene.

**Method:**

We determined the 5'-leader sequences of the dicer mRNAs in human breast cells by 5'-RACE and S1-nuclease protection analysis. We have analyzed the functions of the 5'-leader variants by reporter gene expression *in vitro *and *in vivo*.

**Results:**

We found that the dicer transcripts in human breast cells vary in the sequence of their 5'-leader sequences, and that alternative promoter selection along with alternative splicing of the 5'-terminal exons apparently generate these variations. The breast cell has at least two predominant forms of dicer mRNAs, one of which has an additional 110 nucleotides at the 5'-end. Sequence comparison revealed that the first 80 nucleotides of these mRNA isoforms are encoded by a new exon located approximately 16 kb upstream of the reported start site. There are 30 extra nucleotides added to the previously reported exon 1. The human breast cells studied predominantly express two 5'-leader variants of dicer mRNAs, one with the exons 2 and 3 (long form) and the other without them (short form). By reporter gene expression analysis we found that the exon 2 and 3 sequences at the 5'-leader sequences are greatly inhibitory for the translation of the mRNA into protein.

**Conclusion:**

Dicer gene expression in human breast cells is regulated by alternative promoter selection to alter the length and composition of the 5'-leader sequence of its mRNA. Furthermore, alternative splicing of its exon 2 and 3 sequences of their pre-mRNA creates a more translationally competent mRNA in these cells.

## Introduction

RNA interference (RNAi), a process of silencing gene expression, involves the generation of short, double-stranded RNA (dsRNA) molecules by an enzyme called dicer, which cleaves RNA duplexes into 21–23 base-pair oligomers [1-8]. These oligomers are called, depending on their end-point functions, small interfering RNAs (siRNA), microRNA (miRNA) or short temporal RNA (stRNA) [9]. These small RNA molecules cause sequence-specific post-transcriptional gene silencing by guiding an endonuclease, the RNAi-induced silencing complex (RISC), to mRNA [10,11]. This ubiquitous process has also been recently reported [12-15] in human cells to induce transcriptional silencing through promoter methylation.

Dicer gene expression is regulated in different tissues in humans [16]. Because dicer catalyzes the biosynthesis of miRNAs and stRNAs that in turn regulate the expression of many genes, it is likely that the expression of the dicer gene itself is a highly regulated process [9-11]. While studying the published 5'-leader sequences of human dicer transcripts we noticed that it is infested with many upstream open reading frames (uORF) and out of frame AUG codons [16]. To evaluate whether the 5'-untranslated region of the dicer transcript is in part responsible for the regulation of the dicer transcripts in human breast cells we have amplified, cloned and functionally characterized the 5'-leader sequences of human dicer transcripts from these cells. We report here that the dicer gene in breast cells is transcribed from a far upstream promoter in chromosome 14 and the sequence of the 5'-leader sequences determines the translatability of the dicer transcript and is dictated by alternative splicing of the 5'-exons.

## Materials and methods

### Cell culture

We used a series of commercially available human lines of breast cells, including human mammary epithelial (HME) cells (Clonetics, purchased through Fisher Scientific, Pittsburgh, PA, USA), MDA-MB-231, MCF-7, MDA-MB-468, MCF-10A, and BT549. We also used non-breast cells such as HeLa and HepG2. All cells, other than the HME cells, were purchased from American Type Culture Collection (ATCC, Manassas, VA, USA). HMEC cells were grown in medium purchased from Clonetics under their recommended conditions. Human breast carcinoma MDA-MD-231 and MDA-MD-468 cells were maintained in Leibovitz's L-15 medium supplemented with 1% antibiotic/antimycotic and 10% fetal bovine serum. MCF-10A cells were maintained in a 1:1 mixture of Ham's F12 medium, Dulbecco's modified Eagle's medium supplemented with 1% antibiotic/antimycotic, 0.098 mg/ml cholera toxin, 0.02 μg/μl epidermal growth factor, 0.5 μg/ml hydrocortisone, and 10% horse donor heard serum. BT-549 cells were maintained following standard ATCC recommended media. All cells were maintained in a humidified CO2 (5%) incubator at 37°C. Other cells were maintained and grown in ATCC recommended media and conditions [17,18].

### 5'-RACE

Complementary DNAs (cDNA) were made from total RNA (5 μg) using random primers following standard protocols [18,19]. The cDNAs were dC-tailed and the 5'-ends of the dicer mRNAs were amplified using a dicer gene specific primer (5'-AGTTGACCAAGAACACCG-3'), and the Abridged Anchor Primer (AAP, Invitrogen, Carlsbad, CA, USA) following 5'-rapid amplification of cDNA ends (RACE) analysis protocols from Invitrogen. Amplicons were re-amplified successively using nested dicer gene specific primers (5'-TGACCAAGAACACCGTCC-3', and 5'-AAATGTCTTCCCTGAGCC-3'), and AUAP and UAP, respectively. All PCRs were done using suggested thermocycler conditions of the 5'-RACE protocol (Invitrogen). 5'-RACE products were cloned in the pCRII-Topo vector following TOPO-TA cloning protocols (Invitrogen) and the nucleotide sequences of the cloned inserts were determined by automated DNA sequencing [19].

### S1-nuclease protection assay

Oligonucleotides that were biotinylated at the 5'-end (5'-CACAGCATGCCCAAGCTT CTGCTCTCAAAATGCTGATTCTAAGTTC-3', and 5'-GCATTTTTGTTCTAGCACAGC TTACCTTCCCACTCGCCTGCGTTTC-3') spanning the exon 2 and exon 3 boundary of the long 5'-variant and the exon 1 and exon 4 boundary of the short 5'-variant of the dicer mRNA, respectively, were custom synthesized and gel-purified (Invitrogen). The standard S1-nuclease protection protocol was followed [19] with the following modifications: total cellular RNA (10 μg) was co-precipitated with each probe (10 pmol) and hybridized at 65°C for 15 h. S1-nuclease (Promega, Madison, WI, USA) was used at a concentration of 500 units/ml for 90 min at 37°C. Products were analyzed in a 15% TBE-urea gel, electrophoretically transferred to Zeta Probe blotting membrane (BioRad, Hercules, CA. USA) and biotinylated protected probes were detected using the North2South HRP Detection protocol (Pierce, Rockford, IL, USA).

### 5'Leader sequence/*Renilla *luciferase constructs

The long and short form dicer 5'-leader sequences were amplified using the forward primer 5'-GCGGAAGTGGGTGTTTGTTATTTCC-3' and the reverse primer 5'-GGATCATAAACTTTCGAAGTCATTGCATTTTTGTTCTAGCACAGC-3'. Gene splicing by overlapping extension (SOE) was used to fuse dicer variants to *Renilla *luciferase that was amplified with forward primer 5'-GCTGTGCTAGAACAAAAATGCAATGACTTCGAAAGTTTATGATCC-3' and reverse primer 5'-CTCGAAGCGGCCGCTCTAG-3' [20]. The *Renilla *luciferase ORF alone was amplified using forward primer 5'-ATGACTTCGAAAGTTTATGATCC-3' and reverse primer 5'-CTCGAAGCGGCCGCTCTAG-3'. SOEing products were then ligated into the pCRII-Topo vector (Invitrogen), digested with *Eco*RI (Promega) and cloned at the *Eco*RI site of pcDNA3.1(+) (Invitrogen) and sequenced with T7 primer to determine the orientation of the cloned insert. It is anticipated from the vector information (Invitrogen) that the *Renilla *luciferase transcript from these constructs will have a 133 nucleotide vector derived sequence before the 5'-leader sequences from the dicer gene at their 5'-end. Thus, the nucleotide sequence that is common to the control and the experimental transcripts is 5'-TAGAGAACCCACTGCTTACTGGCTTATCGAAATTAATACGACTCACTATAGGGAGACCCAAGCTGGCTAGCGTTTAAACTTAAGCTTGGTACCGAGCTCGGATCCACTAGTCCAGTGTGGTGGAATTCGGCTT-3'. This sequence does not have any ATG codon.

### Transfection and luciferase assay

Cells were seeded at 80% confluency in a 24-well plate for 24 h in their growth media before co-transfection with one of the pcDNA3.1(+) test plasmid constructs and pGL3-control plasmid. The latter was used as a transfection normalization control. Plasmids were mixed at 0.5 μg per well and transfection was done using the Lipofectamine Plus transfection reagents (Invitrogen) using the protocol suggested by the supplier. After 20 h of incubation in complete medium at 37°C, the cells were lysed in 100 μl passive lysis buffer (Promega) and 5–20 μl of the lysate was assayed [18] for firefly luciferase as well as *Renilla *luciferase activities using Dual Luciferase Assay Reagents (Promega) following suggested protocols [18]. *Renilla *luciferase activity was normalized with respect to firefly luciferase activity and presented as a ratio (relative light units). Protein contents of the extract, when needed, were determined using RC-DC reagents and protocol from BioRad Laboratories Hercules, CA, USA [18].

### *In vitro *transcription and translation

The pcDNA3.1(+) constructs containing the cloned inserts also have a T7 RNA polymerase promoter (Invitrogen). To evaluate whether there is any effect of the dicer 5'-leader sequence insert at the *Eco*RI site in proper orientation on the translatability of the mRNA, we used the T7 RNA polymerase/rabbit reticulocyte lysate *in vitro *transcription/translation system (TNT, Promega). Each plasmid DNA (1 μg) was added to 40 μl of TNT Quick Master Mix containing 1 mM of methionine (Promega). The reactions were incubated at 30°C for 75 min then cooled to 4°C. The TNT reactions were diluted 1:2 with 1x passive lysis buffer (Promega) and incubated at room temperature for 15 min. An aliquot (5–20 μl) was assayed in 100 μl of *Renilla *Luciferase Assay Substrate (Promega).

### Statistical procedures

Each data set is presented as mean ± SEM (N = 12). Statistical significance of a difference between two series of data was tested by determining the *P *value [21]. If the *P *value was less than 0.05, the difference was considered to be significant [21].

## Results and discussion

### Dicer mRNA has a new exon at the 5'-end

**Figure 1:**
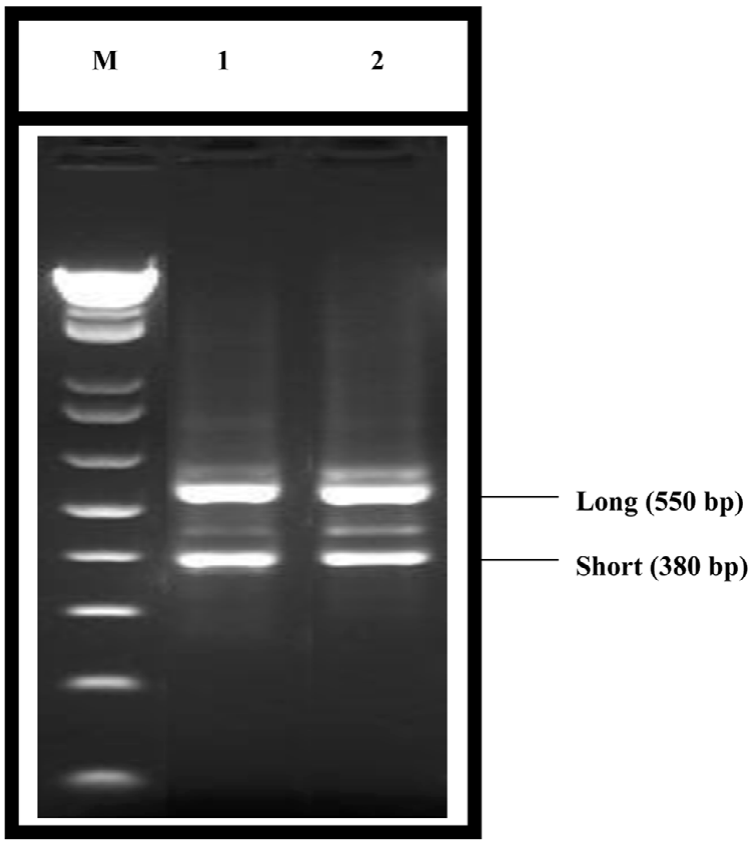
Ethidium bromide stained agarose gel showing the analysis of the 5'-RACE products from human dicer mRNAs from breast cells. Lane M, 1 kb plus DNA ladder (Invitrogen); lane 1, cDNA from MDA-MB-231; lane 2, cDNA from MDA-MB-468. The band at approximately 550 bp is for the unspliced long form of the 5'-leader sequence. The band at approximately 380 bp is the short spliced form.

**Figure 2:**
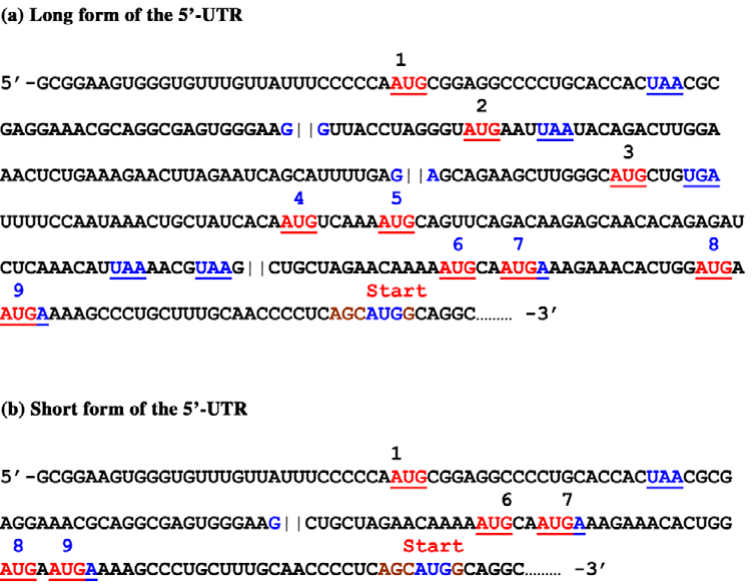
Nucleotide sequences of the **(a) **long form and **(b) **short form of the 5'-leaders derived from the 5'-RACE products (Entrez Accession numbers AY845867 and AY845868). The exon junctions are indicated by two vertical lines. The upstream AUGs and corresponding stop codons are numbered and underscored. The AUG at the 3'-end is the putative start codon.

**Figure 3:**
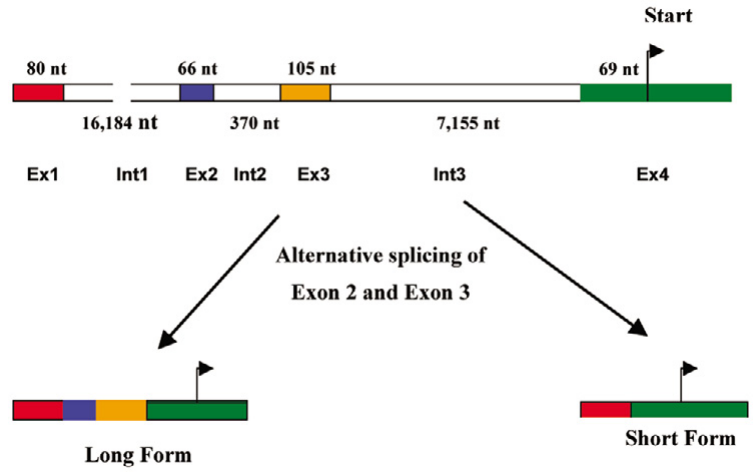
Cartoon showing the possible splicing of the 5'-exons of the human dicer pre-mRNA to produce the short and long forms of mRNAs.

The expression of the dicer gene is differentially regulated in many organisms that have the RNAi mechanism [22-26]. In humans the expression of this gene is highly tissue specific [16] and the mechanisms that regulate it are not known. Initial characterization of dicer mRNA indicated an abundance of overlapping and non-overlapping uORFs at the 5'-leader sequences [16]. To evaluate whether the 5'-leader sequences of dicer mRNA may be a regulatory factor for dicer gene expression, we amplified, cloned and characterized the 5'-leader sequences of these mRNAs from human breast cells. We found that dicer mRNAs have at least two major 5'-leader sequences (Figs 1, 2, 3). Fig. 1 shows our 5'-RACE data from MDA-MB-231 and MDA-MB-468 cells. Similar experiments were done with BT549, MCF7, MCF10A and HMEC cells with similar results (not shown). In fact, we also found these two major forms in HepG2 and HeLa cells (data not shown). We purified the PCR products as a whole and cloned all the DNA molecules. Nucleotide sequencing of more than 40 clones revealed only two types of sequences from dicer mRNAs (Fig. 2). The other bands in the 5'-RACE products were thus PCR artifacts deriving from mis-priming. The 5'-leader sequences in the characterized dicer mRNAs are termed 'long' (320 nucleotides) and 'short' (149 nucleotides) forms because of their lengths. The human dicer gene was originally cloned from HepG2 cells and was shown to be located on chromosome 14q31 near marker D14S605 [16]. The gene was reported to have 28 exons and 27 introns [16]. Our characterization of the 5'-leader sequences of the dicer mRNA variants from breast cells indicate a new exon at the 5'-end and 30 extra nucleotides added to the original first exon (Figs 1, 2, 3). Alignment of the nucleotide sequence to the chromosome 14 sequence shows that the new exon is transcribed from a segment of the chromosome approximately 16 kb upstream of the transcription start site previously predicted for human dicer mRNA. Because we have not done extensive studies with different non-breast cell types, we cannot say at this point that the transcript originally reported for HepG2 or other types of cells does not exist. It is possible that human dicer gene is transcribed from different promoters in different tissues. Selection of this alternative promoter in breast cells leads to the formation of a new intron 1 that is 16,184 base pairs in length (Fig. 3). Alternative promoter selection is a known mode of regulation of the expression of mammalian genes [27-29].

The long form of the 5'-leader sequences contains nine upstream AUG codons (Fig. 2). The short form of the 5'-leader sequence is the alternatively spliced form of the long form. In the short form exon 1 is directly joined to exon 4 and exons 2 and 3 are spliced out (Figs 2 and 3); the number of upstream AUG codons is decreased to five due to this alternative splicing (Fig. 2). Upstream AUG codons often are reported to slow down the rate of translation of an mRNA [30-37]. We tested whether the decrease in the upstream AUGs has any effect on the translatability of the dicer mRNAs (see below).

**Figure 4:**
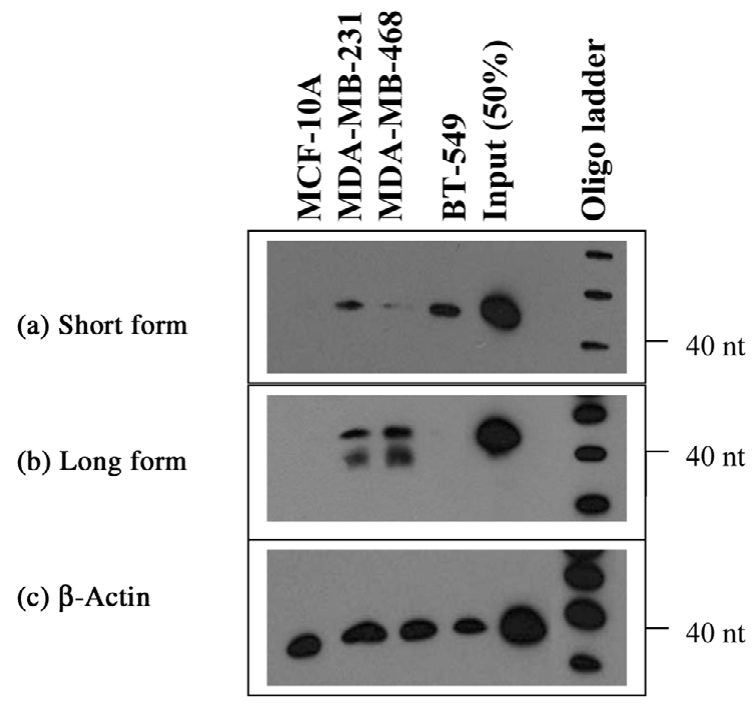
S1-Nuclease protection analysis to verify the long and short forms of dicer mRNA. Autoradiograms showing the relative quantities of **(a) **short from, **(b) **long form and **(c) **β-actin mRNAs. See text for details. Lanes from left to right: RNA from MCF-10A cells; RNA from MDA-MB-231 cells; RNA from MDA-MB-468 cells; RNA from BT-549 cells; 50% of the probe input; and biotinylated oligonucleotide ladder. The lower band in the long form second and third lane from left may be the 3'-truncated (2 nt) form of the biotinylated probe due to the possible degradation of the fraying 3'-end of probe by S1 nuclease.

To ensure that the existence of the different 5'-leader sequences in dicer mRNA as revealed by the 5'-RACE analysis was not a PCR artifact, and also to determine relative quantities of each of the mRNA isoforms, we verified the levels of each of the isoforms by S1-nuclease protection assays (Fig. 4). Data from RNAs isolated from MCF-10A, MDA-MB-231, MDA-MB-468 and BT-549 cells are shown in Fig. 4. We used beta-actin RNA level as a loading control. Both the short and long 5'-leader sequence variants were found in these breast cell lines (not shown). The relative amounts of the two forms vary from cell to cell, indicating that alternative splicing of dicer pre-mRNA could be a form of regulation of dicer gene expression.

### Translation of the short 5'-leader form of the dicer mRNA is more efficient

**Figure 5:**
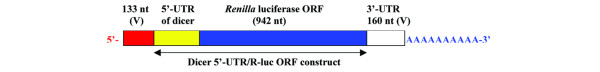
The map of the putative luciferase mRNA synthesized from the plasmid inside the transfected cells. The 5'-end 133 nt come from the vector. The lengths of the dicer 5'-leader sequence vary. The control does not have any insert. The 3'-untranslated region also comes from the vector. Nt, nucleotides; UTR, untranslated region.

**Figure 6:**
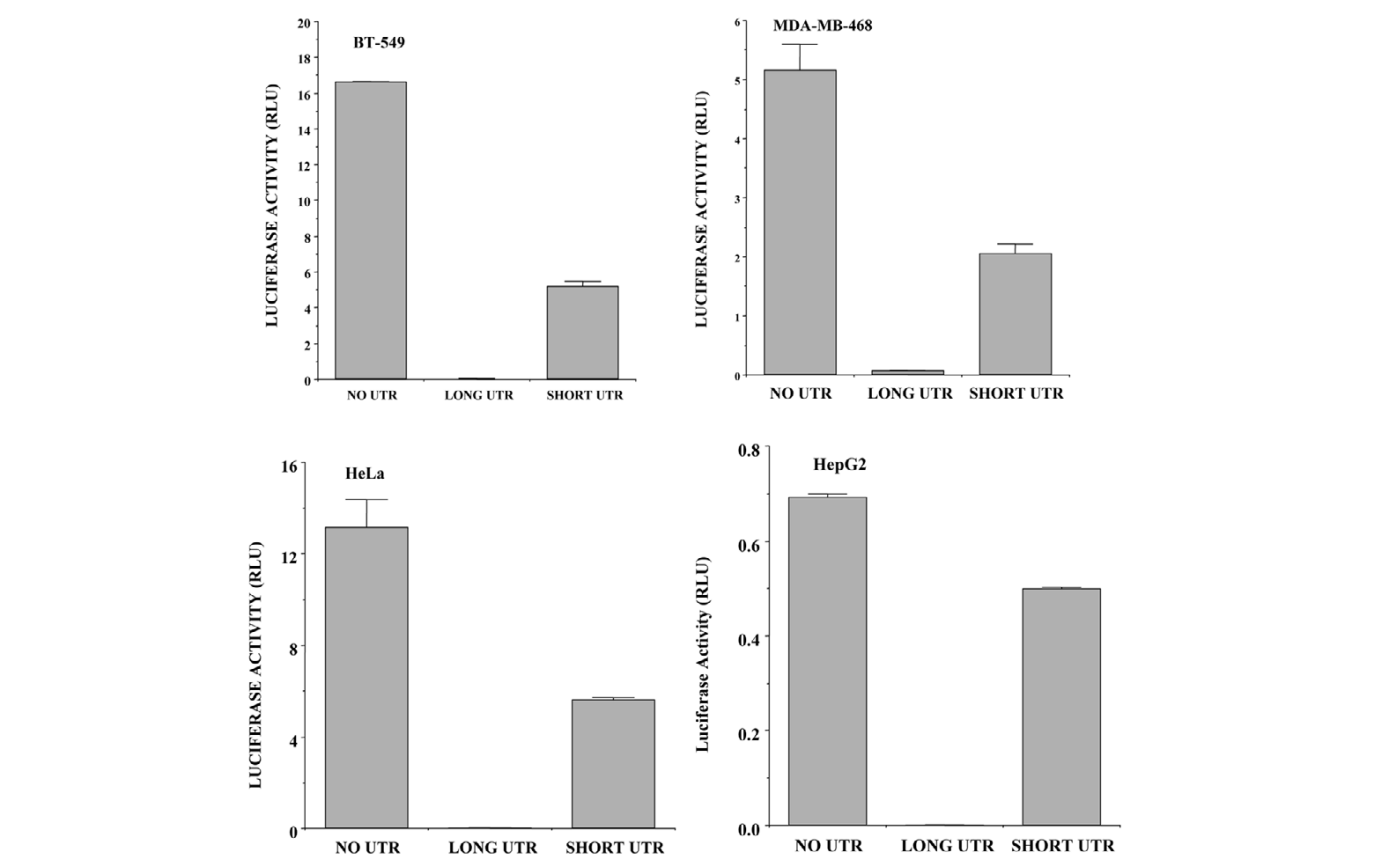
Effect of dicer 5'-leader sequences upstream of the luciferase coding sequence on the expression of relative luciferase activities in BT549, MDA-MB-468, HeLa and HepG2 cells. Luciferase activity is expressed as relative light units (RLU) after normalization with firefly luciferase activity. Results are means of data from 12 different experiments ± standard errors. The differences were statistically significant (p < 0.001). UTR, untranslated region.

**Figure 7:**
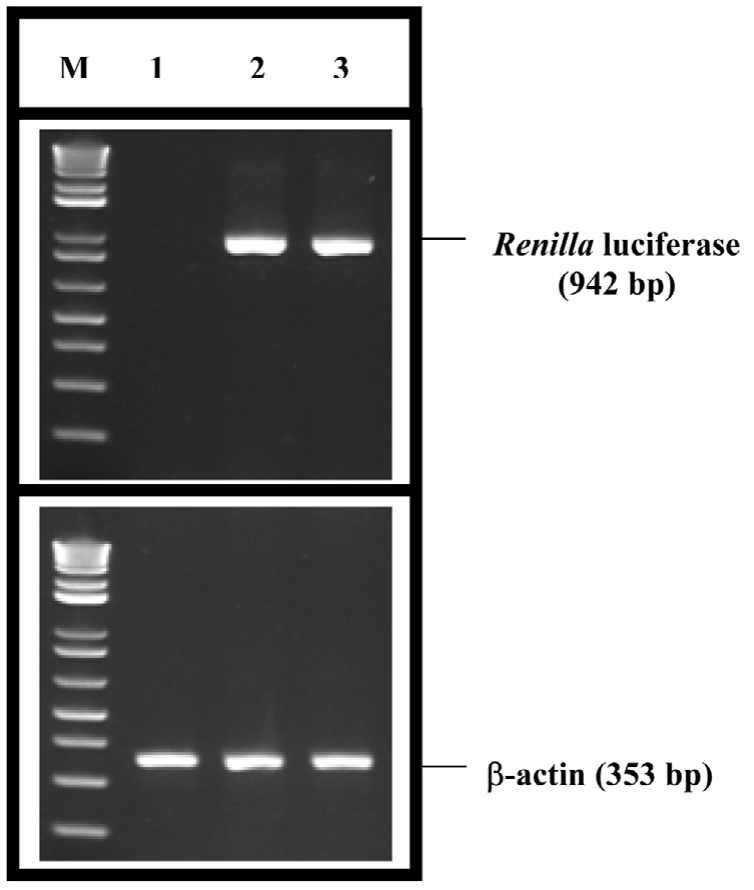
Verification of *Renilla *luciferase mRNA levels by RT-PCR in transfected BT549 cells. β-Actin mRNA levels were used as control. Lane M, 1 kb plus DNA ladder (Invitrogen; lowest band is 200 bp); lane 1–3, RNA isolated from BT549 cells transfected with pcDNA3.1(+), pcDNA3.1(+)-short form 5'-leader or pcDNA3.1(+)-long form plasmid DNA, respectively.

**Figure 8:**
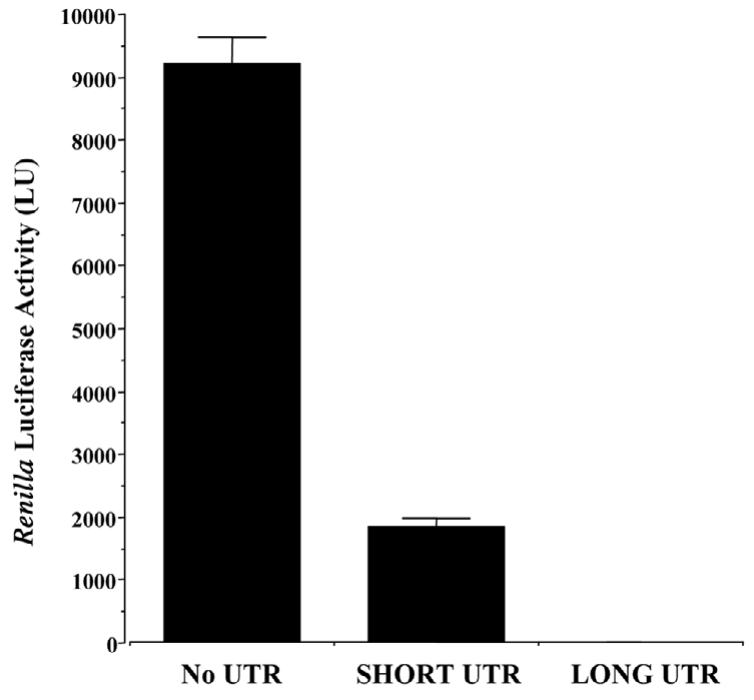
*In vitro *transcription and translation of the *Renilla *luciferase gene from the plasmid constructs and evaluation of the luciferase activity. Results are means of data from 12 different experiments ± standard errors. The differences were statistically significant (p < 0.001). UTR, untranslated region.

To evaluate whether the altered length of the 5'-leader sequences has any effect on the ability of the mRNA to be translated we tested reporter gene expression from constructs that had either the short or the long 5'-leader sequence (Fig. 5). The data shown are with the control constructs, which have no added 5'-leader sequence. We also performed additional experiments with constructs that have other unrelated DNA segments of similar lengths. We found no significant differences between these controls (data not shown). Transient transfection of the breast or non-breast cells with the plasmid constructs was supposed to produce *Renilla *luciferase mRNAs with the general structure shown in Fig. 5. We lysed the transfected cells after 20 h and assayed for *Renilla *luciferase activity in the cell lysate. The plasmid that constitutively expresses firefly luciferase from SV40 late promoter was used as transfection control and the *Renilla *luciferase data were normalized with respect to the firefly luciferase activity in the extract. Tthe construct with the long form of the 5'-leader sequence expressed no significant *Renilla *luciferase activity compared to the controls (Fig. 6) in all the cells tested. On the other hand, the construct with the short form of the 5'-leader sequence expressed significant *Renilla *luciferase activity (Fig. 6). The activity from the construct with the short form of the 5'-leader sequence was much less than from the constructs with no added 5'-leader sequence, indicating that the residual AUG codons in the short form of the 5'-leader sequence may still be negatively regulating the translation and/or stability of the mRNA. To verify whether inclusion of different 5'-leader sequences in the reporter plasmid has any effect on the level of *Renilla *luciferase mRNA, we performed RT-PCR analysis. Our data suggest that irrespective of whether the construct expresses luciferase activity, there is no significant difference in the luciferase mRNA levels (Fig. 7). The effect of the added 5'-leader sequences thus may be at the translational level. We further verified this by *in vitro *transcription/translation with the plasmid constructs followed by reporter gene expression analysis (Fig. 8). This experiment also suggests that perhaps the consequence of altering the 5'-leader sequence of human dicer transcripts is the altered translation of the mRNA into dicer protein. Taken together, these observations imply that alternative promoter use and alternative splicing of 5'-exons are important for the regulation of human dicer gene expression.

## Conclusion

In human breast cells, the dicer gene is transcribed from a promoter that is more than 16 kbp upstream of the initiation site reported for this gene from non-breast cells [16]. This alternative promoter selection modifies the length and composition of the 5'-leader sequences of its mRNA. Furthermore, alternative splicing of the exon 2 and 3 sequences of its pre-mRNA creates a more translationally competent mRNA in these cells. Breast cell dicer mRNAs have a high number of upstream AUG codons with in frame stop codons. Translational efficiency and/or stability of the RNA are decreased due to this type of 5'-leader sequence [34-37]. The non-sense mediated degradation pathway of mRNA decay may also be stimulated by these uORFs [38,39]. It is interesting to note that there is an association between active dicer mRNA expression and the invasiveness of the breast cell line. Recently we found that the zinc finger repressor protein SLUG may have the determinative role in the invasiveness of the breast cell [40,41]. We are exploring whether dicer regulates SLUG gene expression in human breast cells. The mechanisms by which the 5'-leader sequences of the dicer mRNAs in breast cells regulate the expression of this gene are yet to be determined.

## Abbreviations

ATCC = American Type Culture Collection; dsRNA = double-stranded RNA; HME = human mammary epithelial; kb = kilobases; miRNA = microRNA; nt = nucleotide; PCR = polymerase chain reaction; RACE = rapid amplification of cDNA ends; RISC = RNAi-induced silencing complex; RNAi = RNA interference; siRNA = small interfering RNA; SOE = splicing by overlapping extension; stRNA = short temporal RNA; uORF = upstream open reading frame.

## Competing interests

The author(s) declare that they have no competing interests.

## Authors' contributions

CVIW executed the experiments described in the manuscript and wrote the initial drafts of the manuscript. GC conceived and coordinated the study. All authors read and approved the final manuscript.
